# Unveiling the microbial influence: bacteria’s dual role in tumor metastasis

**DOI:** 10.3389/fonc.2025.1524887

**Published:** 2025-03-14

**Authors:** Liying Lin, Dongyan Zhang

**Affiliations:** Department of Precision Biomedical Key Laboratory, Department of Stomatology, Liaocheng People’s Hospital, Shandong Provincial Key Medical and Health Laboratory of Precision Medicine for Aging Intervention and Active Health, Liaocheng, China

**Keywords:** tumor metastasis, bacteria, chronic inflammation, immune escape, ECM remodeling, immunotherapy, antibiotics

## Abstract

As cancer research advances, the intricate relationship between the microbiome and cancer is gaining heightened recognition, especially concerning tumor metastasis, where bacterial involvement becomes increasingly complex. This review seeks to systematically examine the dual roles of bacteria in the tumor metastasis process, encompassing both mechanisms that facilitate metastasis and the inhibitory effects exerted by specific microorganisms. We explore the mechanisms through which bacteria influence tumor cell migration by inducing chronic inflammation, evading host immune responses, and remodeling the ECM. Moreover, the immunomodulatory potential of probiotics and genetically engineered bacteria offers promising prospects for the prevention and treatment of tumor metastasis. This article elucidates the complexity and emerging frontiers of bacterial involvement in tumor metastasis by examining the clinical significance of bacteria as potential biomarkers and evaluating the effects of antibiotic usage on the metastatic process. We posit that comprehending the biological characteristics and clinical significance of bacteria, as a critical component of the tumor microenvironment, will offer innovative strategies and theoretical foundations for cancer treatment. Furthermore, this article explores future research directions, including the application of microbiome technologies and bacteria-based therapeutic strategies, thereby offering a valuable perspective for the development of novel anti-cancer approaches.

## 1 Introduction

Cancer metastasis constitutes a principal cause of cancer-related mortality, characterized by the invasion of adjacent tissues by cancer cells originating from the primary tumor site, followed by their dissemination through the circulatory or lymphatic systems to distant organs (1). Although extensive research has elucidated the molecular mechanisms and cellular behaviors implicated in tumor metastasis, the microenvironmental factors influencing this process remain inadequately understood and warrant further investigation (1, 2). Recent research on the relationship between the microbiome and cancer has progressively revealed novel aspects of tumor biology, with particular emphasis on the role of bacteria in tumor metastasis, which has garnered significant attention (3). Bacteria constitute a crucial component of the tumor microenvironment; these microorganisms are not only present in the intestinal tract but are also widely distributed across the microbial communities of the oral cavity, cervix, and other organs (4).

Bacteria exert significant influence on the host immune system through diverse mechanisms and are integral to the initiation, progression, and metastasis of tumors. Research has identified associations between genetic predispositions within the gut microbiome and the incidence of various cancers, including breast, lung, and oral cancers (5). Dysbiosis, or imbalances in gut microbiota, can precipitate chronic inflammation, a recognized risk factor for several cancers, notably colorectal cancer (CRC) (6, 7). Certain bacteria, such as clostridium nucleatum, can directly impact the tumor microenvironment by modulating immune cell infiltration and activity (8). Tumor-associated bacteria have the potential to foster a pre-tumorigenic immune milieu, thereby facilitating tumor progression and metastasis (9). Conversely, other microorganisms, such as those within the Lachnospiraceae family, may exert inhibitory effects by eliciting anti-tumor immune responses (10).

A comprehensive investigation into the role of bacteria in tumor metastasis is expected to elucidate the intricate mechanisms underlying metastatic processes and potentially offer novel insights for clinical interventions. Notably, specific bacterial species have been identified as significantly associated with particular tumor types, exemplified by the correlation between Helicobacter pylori and gastric cancer (11), as well as the dysregulation of the intestinal microbiome in CRC (12, 13). More significantly, the composition and diversity of bacterial populations may play a crucial role in influencing the metastatic potential of tumors, thereby presenting novel opportunities for the development of bacterial-based biomarkers (14). Furthermore, bacteria and their metabolites may serve as promising targets for immunotherapy, potentially enhancing the efficacy of existing anti-tumor treatments. Nevertheless, our current understanding of the dual role of bacteria in tumor metastasis remains limited.

This review aims to examine the mechanisms by which bacteria promote or inhibit tumor metastasis, assess their clinical significance, and suggest directions for future research. We will investigate the influence of bacteria on the metastatic process through their roles in inducing inflammation, modifying immune responses, and reshaping the ECM. Additionally, we will explore the potential of bacteria as biomarkers and novel therapeutic strategies. Through this comprehensive analysis, we intend to offer new insights into the study of tumor metastasis, thereby paving the way for the development of innovative approaches in this field.

## 2 Biological mechanisms of bacteria in tumor metastasis

### 2.1 The role of Bacterial in chronic inflammation and immune modulation

Research indicates that bacterial infections can facilitate the initiation and progression of tumors through multiple mechanisms. For instance, bacteria can induce a chronic inflammatory response, resulting in the persistent activation of the immune system. This not only promotes tumor cell proliferation but may also enhance metastasis by modifying the tumor microenvironment (15). Specifically, bacterial infections lead to the infiltration of immune cells into the tumor microenvironment, particularly the accumulation of macrophages and neutrophils, which exhibit a dual role in tumor progression by both inhibiting tumor growth and promoting metastasis (16). Certain studies have demonstrated that chronic lung infections caused by Pseudomonas aeruginosa can recruit neutrophils with highly expression of major histocompatibility complex class II, thereby altering the pulmonary microenvironment and facilitating lung metastasis of breast cancer (BC) (17). Additionally, bacterial infections may activate host inflammatory signaling pathways by releasing bacterial products, such as endotoxins, which promote tumor cell migration and invasion (18, 19). It has been observed that enterotoxigenic Bacteroides fragilis colonization in breast ducts may induce tumor cell growth and metastasis through the β-catenin and Notch1 pathways (20).

Certain bacterial species have the capacity to enhance the proliferation of regulatory T cells through the production of immunosuppressive factors, thereby attenuating the anti-tumor immune response (21). Notably, Campylobacter is markedly enriched in metastatic lesions of primary CRC, and its cytolethal distending toxin has been implicated in promoting CRC metastasis via the JAK2-STAT3-MMP9 signaling pathway in murine models of hepatic or pulmonary metastasis (22). Empirical evidence underscores the pivotal role of the gut microbiota in modulating host immune responses, with specific beneficial bacteria, such as Akkermansia muciniphila and Bifidobacterium longum, being identified as enhancers of anti-tumor immunity (23, 24). In the context of colon cancer, dysregulation of the intestinal microbiota occurs, wherein bacterial inflammatory factors can facilitate tumorigenesis and metastasis by activating NF-κB and other pro-inflammatory signaling pathways (25). Furthermore, certain tumor cells, including melanoma, can exploit metabolites produced by intestinal bacteria to modulate their immune evasion mechanisms, thereby augmenting their survival (26). In addition, bacteria have the capability to influence immune cell function through the production of extracellular vesicles, which may transport tumor antigens or immunosuppressive factors, thereby facilitating immune evasion (27, 28). Research has demonstrated that Fusobacterium nucleatum can enhance cancer growth and metastasis by releasing toll-like receptor 4 (TLR4), which interacts with BC cells via extracellular vesicles (29). Conversely, Lactobacillus casei and Lactobacillus reuteri have been shown to inhibit the proliferation and migration of pancreatic cancer cells by suppressing TLR4. These bacteria also impede the induction of M2 macrophages by pancreatic cancer cells and promote the differentiation of M1 macrophages (30).

### 2.2 Bacterial and tumor metastasis niches

Bacteria are integral to the establishment of niches at sites of tumor metastasis. Research has demonstrated the presence of viable bacteria within murine breast tumors, which preemptively migrate to the lungs to facilitate the metastasis of BC cells. Notably, the removal of these bacteria does not impact the growth of the primary tumor but significantly reduces lung metastasis (31). Similarly, Escherichia coli residing in CRC disrupts the gut vascular barrier and metastasizes to the liver, thereby promoting the formation of pre-metastatic niches and facilitating the metastasis of CRC cells (32). These findings offer novel insights and potential therapeutic targets for the development of innovative anti-tumor metastasis strategies.

### 2.3 Bacterial-induced ECM remodeling

The ECM constitutes a critical element of the tumor microenvironment, serving not only as a structural scaffold for tumor cells but also modulating their proliferation and metastatic potential through interactions with these cells.

Bacteria possess the ability to degrade ECM components through the secretion of autocrine or induced enzymes, consequently modifying the physical and chemical properties of the tumor microenvironment. For example, Helicobacter pylori can stimulate the secretion of cellular matrix metalloproteinase (MMPs) to degrade collagen and other ECM components via MAPK signaling pathways, a mechanism that may facilitate tumor cell migration and enhance tumor aggressiveness (33). Moreover, the introduction of excessive Escherichia coli in antibiotic-treated murine models of CRC has been observed to upregulate cathepsin K expression in tumors, a factor associated with increased liver metastasis (34). Additionally, bacterial presence can trigger local inflammatory responses, thereby promoting ECM remodeling. Lourdes et al. provided a comprehensive analysis demonstrating that the infiltration of inflammatory cells induces the rearrangement and synthesis of ECM components in BC, thereby creating a microenvironment conducive to tumor growth (35). Furthermore, bacterial prostatitis is associated with ERG^+^ pre-cancerous changes, suggesting that bacterial infection may activate oncogenic driver genes in prostate tissue, a process that involves modifications to the ECM (36).

In recent years, bacteria-based cancer therapies targeting the ECM have demonstrated significant potential. One study revealed that engineered Salmonella typhimurium could degrade the ECM within tumors by secreting the HysA protein, thereby enhancing the delivery of chemotherapeutic agents (37). Another study introduced the engineered bacterium VNP^NKase^, which was designed to sustainably degrade the ECM, inactivate cancer-associated fibroblasts, and facilitate the recruitment of dendritic cells and T cells (38). Although the application of bacteria and ECM in tumor therapy remains in its nascent stages, initial investigations have highlighted the promise of this approach. Future research is expected to advance the field by exploring more effective treatment modalities and underlying mechanisms. It is crucial to emphasize that ensuring the safety and efficacy of bacterial therapies, along with understanding their impact on the host immune system, is essential for the development of clinical applications.

**Figure 1:**
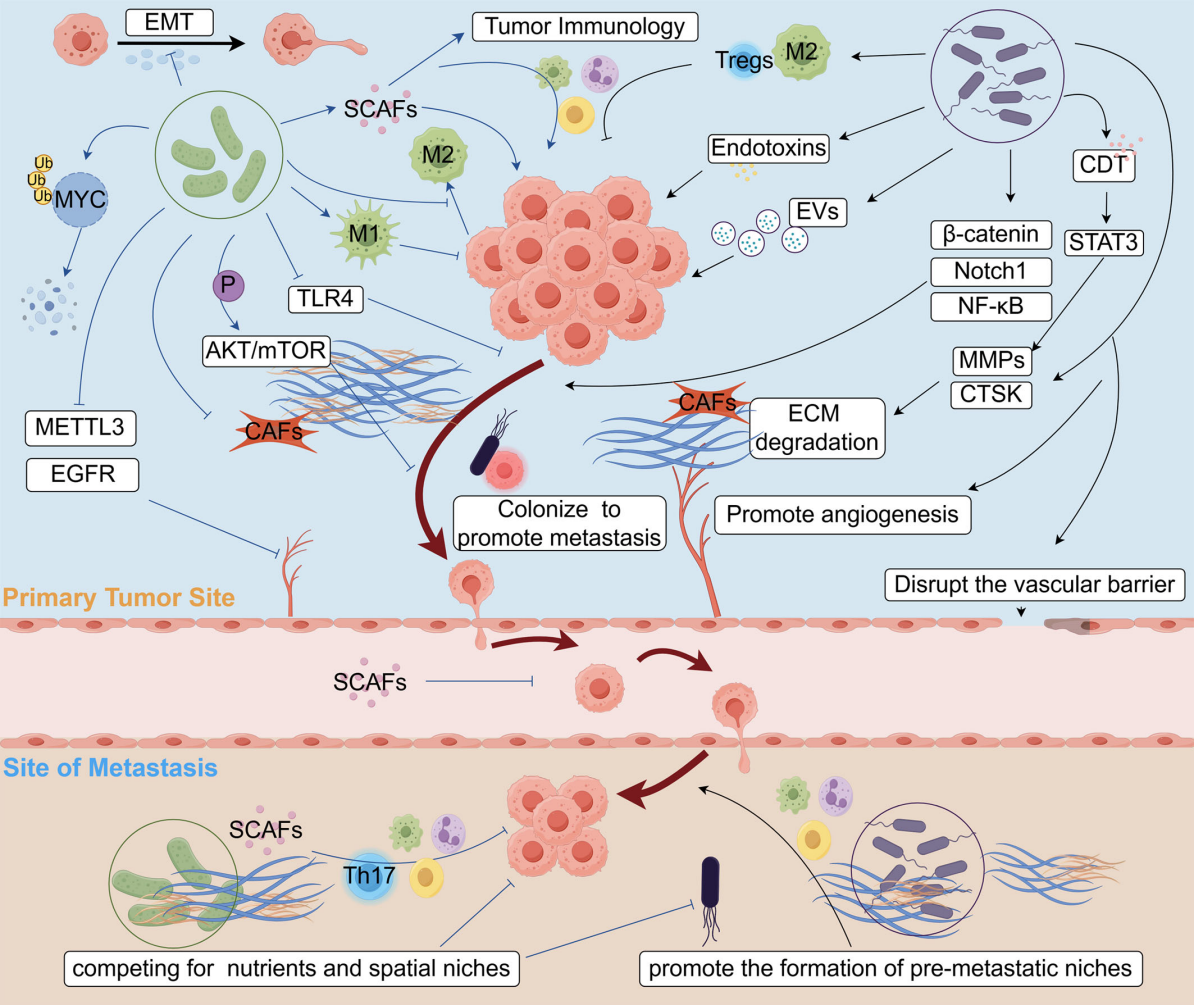
Biological mechanisms of bacteria in tumor metastasis.

The intricate interplay between bacteria-induced chronic inflammation and the ECM remodeling is characterized by a series of complex interactions. Chronic infection triggers the activation of the immune system, resulting in the release of inflammatory mediators and an upregulation of MMPs, which subsequently lead to the degradation and remodeling of ECM components. This remodeling process alters the tissue and tumor microenvironment, thereby facilitating tumor cell migration and invasion and elevating the risk of cancer metastasis (**Figure 1**, by figdraw.com, UWSSOc4e14). A comprehensive investigation of this mechanism is crucial not only for enhancing our understanding of the pathological processes underlying related diseases but also for potentially providing valuable insights for the development of innovative therapeutic strategies.

## 3 Bacterial inhibition and its effect on metastasis

### 3.1 Probiotics that inhibit metastasis

Research indicates that certain bacterial species can facilitate tumor metastasis, whereas others can inhibit this process by activating the host’s immune response, thereby enhancing the efficacy of cancer immunotherapy (**Tables 1**, **2**). Notably, lactic acid bacteria have demonstrated significant potential in suppressing tumor metastasis (39). A study investigated the application of Veillonella parvula in treating BC in mice, revealing a reduction in mean tumor volume and liver metastases by 44.3% and 51.6%, respectively. This effect may be attributed to the decreased lactic acid levels in the tumor microenvironment (40). Additionally, milk fermented with Lactobacillus casei CRL431 was found to mitigate capecitabine-induced side effects and reduce tumor metastasis across various mouse models of BC (41). Furthermore, while Porphyromonas gingivalis has been shown to promote pancreatic intraepithelial neoplasia (PanIN), Lactobacillus species have been observed to impede the progression of these lesions and inhibit epithelial-mesenchymal transition (EMT) (42).

**Table 1:** Bacteria that promote tumor metastasis.

Bacterial Species	Mechanism	Tumor type	Ref
F. nucleatum	Suppress the accumulation of T cell	BC	(8)
P. aeruginosa	Recruit MHCII ^(hi)^ neutrophils to altering the pulmonary microenvironment and facilitating lung metastasis	BC	(17)
ETBF	Activate Notch and β-Catenin signaling	BC	(20)
Campylobacter	Secret CDT and activate JAK2-STAT3-MMP9 signaling	CRC	(22)
F. nucleatum	Releasing TLR4	BC	(29)
E. coli	Disrupts the gut vascular barrier; Metastasis to the liver and promote the formation of pre-metastatic niches	CRC	(32)
H. pylori	Stimulate the secretion of MMPs via MAPK signaling	SC	(33)
E. coli	Induce the expression of cathepsin K	CRC	(34)
F. nucleatum	Activate CCL20 and M2 macrophages polarization	CRC	(64)
P. gingivalis	Enhances OSCC cell proliferation, promotes angiogenesis, induces EMT	OSCC	(72)

**Table 2:** Probiotics that inhibit tumor metastasis.

Bacterial Species	Mechanism	Tumor type	Ref
L. casei and L. reuteri	Inhibit the proliferation, migration, invasion of pancreatic cancer cells; Inhibit pancreatic cancer cell-induced M2 macrophages by suppressing TLR4 expression	PC	(30)
Veillonella parvula	Decreased lactate level	BC	(40)
L. casei CRL431	Inhibite TGF-β signaling; Reduce the viability of pancreatic cancer cells	BC	(41)
Lactobacillus	Inhibit EMT	PanIN	(42)
C. butyricum	Mediates MYC ubiquitination degradation	CRC	(43)
C. butyricum	Down-regulate METTL3, vimentin and VEGFR2	CRC	(44)
Salmonella	Phosphorylate AKT/mTOR pathway; Down-regulates the expression of adhesion molecules in epithelial cells	B16F10/CT26	(45)
L. casei W56; L. acidophilus W37; L. brevis W63; L. lactis W58; B. lactis W52; L. lactis W19; L. salivarius W24; B. bifidum W23	Reduce tumor angiogenesis	CRC	(46)
Probiotic mixture VSL#3	Upregulate CCL20 in lung endothelial cells; Recruitment Th17	Melanoma	(47)
L. rhamnosus	Competing for nutrition and living space	Melanoma	(49)
L. paracasei	Inhibit the proliferation of CRC cells and promote cell apoptosis	CRC	(57)

A study has demonstrated that Clostridium butyricum can mitigate MYC-mediated resistance to 5-fluorouracil and enhance the efficacy of anti-PD1 immunotherapy by facilitating the ubiquitination and subsequent degradation of MYC. This process ultimately inhibits the proliferation and metastasis of CRC (43). Additionally, research indicates that Clostridium butyrate downregulates the expression of METTL3 in CRC cells, leading to decreased levels of vimentin and VEGFR2. This downregulation consequently reduces EMT and angiogenesis, thereby inhibiting tumor metastasis in nude mice (44). Furthermore, Salmonella has been shown to impede tumor metastasis by downregulating the expression of epithelial cell adhesion molecules via the phosphorylated AKT/mTOR signaling pathway (45).

Mixed probiotics have been employed in research to impede tumor metastasis. In particular, a multi-species probiotic formulation was administered to treat CRC in rats, yielding results that demonstrated a significant reduction in tumor angiogenesis and inhibition of CRC liver metastasis (46). Furthermore, the administration of probiotic supplements in mice with melanoma has been shown to enhance the population of short-chain fatty acid-producing bacteria in the gut, elevate the levels of short-chain fatty acids (SCFAs) in the bloodstream, and promote the recruitment of helper T cell 17 by upregulating the expression of chemokine (C-C motif) ligand 20 (CCL20) in lung endothelial cells. These effects collectively contribute to a reduction in lung metastases (47).

Certain bacterial species exert an inhibitory influence within the tumor microenvironment by competing with tumor cells for essential nutrients and spatial niches. The metabolites produced by these bacteria may directly impede tumor cell proliferation and migration (48). For instance, a study demonstrated enhanced chemotherapy efficacy against advanced melanoma B16 metastasis through the aerosol inhalation of antibiotics (vancomycin/neomycin) or Bacillus rhamnosus, resulting in the inhibition of lung metastasis (49). Furthermore, research has indicated that SCFAs generated by intestinal bacteria, such as valerate and butyrate, can enhance tumor immune efficacy by modulating the immune response (50). Notably, butyrate, a prevalent SCFA, has been shown to inhibit tumor growth in murine models and potentially reverse immunosuppression induced by elevated levels of tumor-associated immune cells, including PD-L1 and IL-10 (51). These findings offer novel insights into the potential application of bacteria and their metabolites as anti-tumor therapeutic strategies.

### 3.2 Bacteria-based immunotherapy strategies

Bacteria-based immunotherapy strategies have garnered significant attention in recent years owing to their promising potential in cancer treatment. Bacteria possess the capability to selectively localize at tumor sites and augment the anti-tumor response by activating the host immune system (52). Intravenous administration of Escherichia coli has been shown to colonize necrotic or hypoxic tumor regions. In a study conducted by Jiang et al., Escherichia coli was utilized as an engineered bacterial carrier in conjunction with radiotherapy, resulting in a significant inhibition of CRC growth and metastasis in murine models (53). The application of engineered bacteria presents considerable potential in the field of bacteria-based immunotherapy. Recent advancements in synthetic biology and nanotechnology have facilitated the development of bacteria with improved tumor specificity, enabling the targeted delivery of therapeutic proteins, cytokines, or drugs directly to tumor sites (54). Certain studies have involved the engineering of Escherichia coli to express human CXCL16, which has been employed in the treatment of various tumor models in mice. These engineered bacteria have the capacity to release chemokines within the tumor microenvironment, thereby attracting an influx of immune cells to the tumor site and augmenting the anti-tumor immune response (55). Bacterial membranes have been extensively investigated as potent adjuvants for tumor vaccines, attributed to their inherent capacity to stimulate the immune system. Several studies have employed nanoparticles encapsulated within double-membrane vesicles derived from the Salmonella strain VNP20009 to treat malignant melanoma tumors in murine models, resulting in a marked enhancement of therapeutic efficacy and the promotion of anti-tumor immunity (56). The incorporation of cancer vaccines within bacterial outer membranes has been shown to effectively activate the murine immune system, facilitating the production of robust immune protection upon the presentation of tumor antigens (57).

The combined application of bacterial therapy and immunotherapy is also an emerging treatment strategy. In a clinical trial involving 20 patients with advanced melanoma who had not previously undergone treatment, a combination therapy of fecal microbiota transplantation (FMT) and PD-1 inhibitors was administered. The FMT treatment facilitated the reduction of harmful bacteria without inducing adverse events (58). Additionally, a study investigating the use of Lactobacillus paracasei and cytokine-induced killer cells in the treatment of CRC in nude mice demonstrated that Lactobacillus paracasei effectively inhibited tumor growth and liver metastasis, with the synergistic treatment yielding superior outcomes (59).

**Figure 2:**
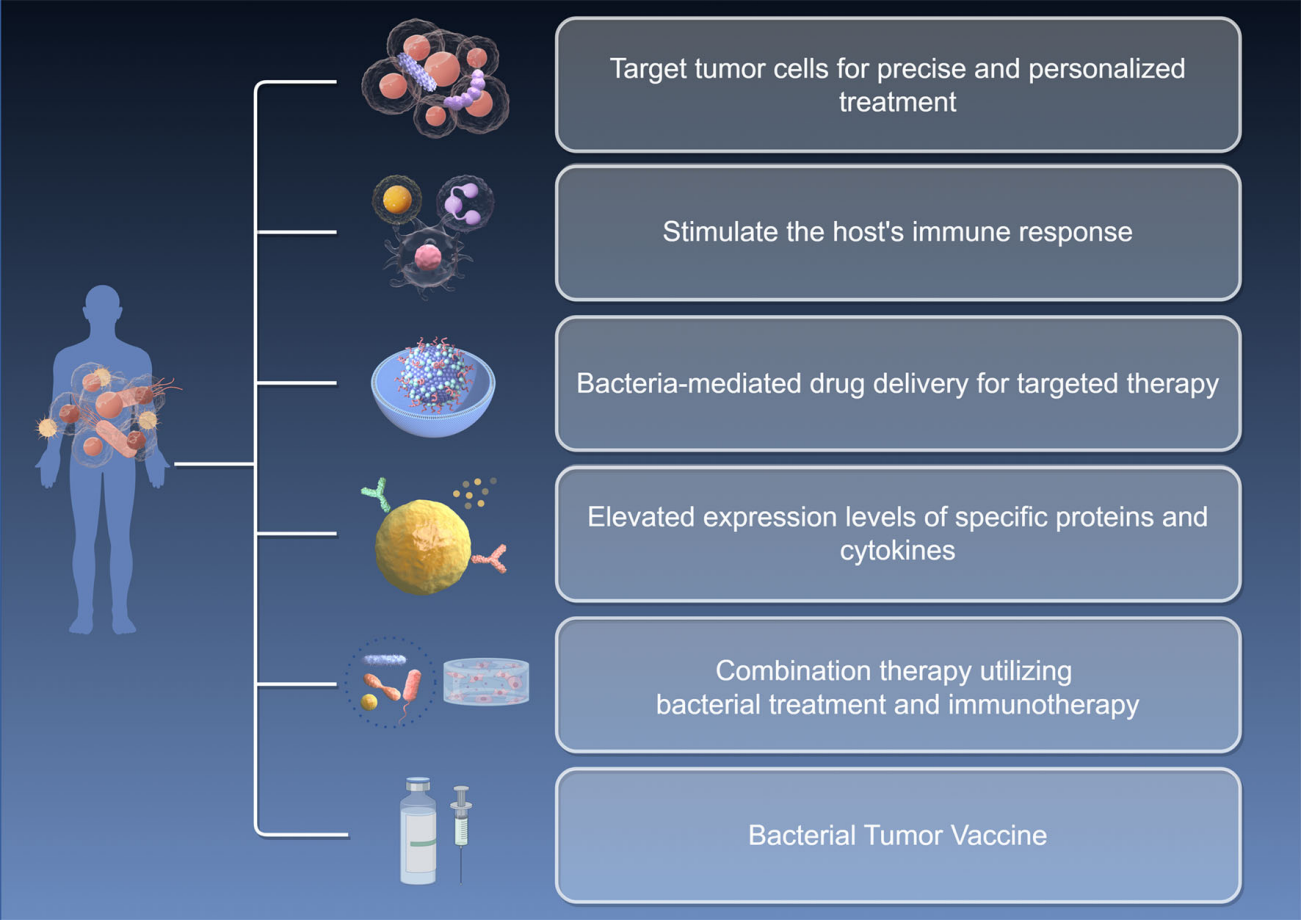
Bacteria-based immunotherapy strategies.

While bacteria-based immunotherapy and probiotic strategies hold significant promise for inhibiting tumor metastasis, several challenges remain, including variability in individual efficacy, as well as concerns regarding the safety and stability of probiotics. To address these issues, future research should prioritize the development of personalized treatment regimens, optimize the screening and application processes for probiotics, and conduct in-depth investigations into their mechanisms of action. Furthermore, integrating these approaches with other immunotherapy and precision medicine strategies may enhance therapeutic outcomes and improve the prognosis for patients with tumors (**Figure 2**, by figdraw.com, SSWPY088bd).

## 4 Clinical correlations between bacteria and tumor metastasis

### 4.1 Bacteria as potential biomarkers in oncology

In recent years, increasing attention has been directed towards the relationship between bacteria and tumor metastasis. Bo et al. discovered that bacteria present in BC cells can accompany the metastasis and colonization of these cells. These bacteria are capable of modulating the actin cytoskeleton of host cells, thereby enhancing the survival of tumor cells in the circulatory system under conditions of fluid shear stress. Furthermore, the removal of tumor-associated microorganisms impedes metastasis, although it does not inhibit the growth of the primary tumor (31). In the tumor microbiome of BC patients, there is an increased infiltration of Bacillus, Pseudomonas, Brevibacterium, and Mycobacterium. Notably, a heightened abundance of Paenibacillus in these patients correlates with a reduced survival rate (60). Furthermore, the composition of the gut microbiota is considered a crucial factor influencing the response to immune checkpoint inhibitors (ICPI), offering novel insights for personalized cancer immunotherapy (61). In CRC research, it was observed that the predominant bacteria in male CRC patients were Bacteroides, Eubacterium, and Faecalibacterium, whereas in female CRC patients, Bacteroides, Subdoligranulum, and Eubacterium were most prevalent (62). A meta-analysis indicated a significant association between F. nucleatum and CRC metastasis (63), with F. nucleatum promoting CRC metastasis via the miR-1322/CCL20 axis and M2 macrophage polarization (64). Additional studies have identified that the dominant bacterial species in periodontitis, Porphyromonas gingivalis, can facilitate the onset and progression of pancreatic cancer in murine models (65). Concurrently, a clinical trial revealed that a low abundance of Eubacterium infirmum, Actinobaculum, and Selenomonas is linked to a poor response in oral squamous cell carcinoma (OSCC) patients undergoing neoadjuvant therapy (66). Variations in bacterial exosomes between healthy individuals and cancer patients may represent a novel biomarker, demonstrating significant diagnostic potential across a range of diseases. The molecular content of bacterial exosomes can provide insights into the host’s health status and may facilitate the early detection of diseases (67).

The aforementioned findings position bacterial profiling as a promising instrument for assessing metastasis risk. Utilizing high-throughput sequencing technology enables a comprehensive analysis of gut microbiota composition, thereby elucidating associations between specific bacterial profiles and tumor metastasis. This analytical approach not only facilitates personalized risk assessments for individual patients but also lays the theoretical groundwork for the development of targeted interventions. As technology continues to advance and improve, bacterial profiling is anticipated to become a standard methodology in clinical practice.

### 4.2 The impact of antibiotics on tumor metastasis

The administration of antibiotics notably influences the risk of metastasis during cancer treatment. While antibiotics can modify the gut microbiome by decreasing the abandance of pathogenic bacteria, they may concurrently disturb the equilibrium of beneficial microbial populations, potentially exerting adverse effects on tumor metastasis. An epidemiological investigation encompassing various tumors, such as non-small cell lung cancer and melanoma, revealed that patients receiving ICPI who were exposed to antibiotics exhibited reduced progression-free survival, overall survival, and response rates (68). Research indicates that the intestinal microbiota plays a role in the treatment of HER2-positive BC with trastuzumab, whereas the administration of antibiotics diminishes the efficacy of this treatment (69). In murine models of metastasis using LLC and B16-F10 cells, SPF mice treated with the antibiotic cocktail (ABX) exhibited increased lung metastasis. Conversely, the transplantation of bifidobacterium into germ-free mice markedly inhibited lung metastasis (70). The administration of ABX can significantly disrupt the balance of intestinal microbiota and exacerbate cancer metastasis in Lewis lung cancer mouse models (71). These findings underscore the critical importance of maintaining microbial ecological balance in cancer therapy and suggest that broad-spectrum antibiotics should be employed with caution in future clinical settings, particularly for patients at risk of tumor progression and metastasis.

A study has demonstrated that porphyromonas gingivalis enhances OSCC cell proliferation, promotes angiogenesis, induces epithelial-to-mesenchymal transition, and generates carcinogenic metabolites. In contrast, oral Streptococcus species, such as Streptococcus gordonii, counteract the pro-tumor epithelial cell phenotype induced by Porphyromonas gingivalis (72). Furthermore, other researchers have identified that Fusobacterium nucleatum utilizes the lectin FAP2 to colonize the surface of BC cells, thereby facilitating tumor growth and metastasis. The application of antibiotics and anti-FAP2 treatments has been shown to mitigate the tumor-promoting effects of F. nucleatum (8). These findings suggest that early targeted bacterial therapies may play a crucial role in preventing metastasis and tumor dissemination. Additionally, research has explored the use of non-absorbable antibiotics to decrease CRC proliferation and metastasis by reducing the population of bacteria responsible for producing deoxycholic acid (DCA) (73). Moreover, salicycin has been found to inhibit tumor growth and metastasis by reversing the immunosuppressive microenvironment (74).

The role of bacteria as biomarkers for tumor metastasis is garnering increasing attention. The presence of specific bacterial species has been linked to the risk of tumor metastasis, offering a novel perspective for early screening and personalized treatment approaches. Concurrently, the administration of antibiotics has the potential to alter the microbial community, which may, in turn, influence the metastatic process. These observations underscore the significant role of microbial ecology in tumor development and treatment. Consequently, future research should focus on elucidating the interactions between bacteria and tumors to optimize anti-tumor strategies and enhance treatment efficacy.

## 5 Frontier research and future direction

### 5.1 Integrated study of microbiome and tumor metastasis

The advent of high-throughput sequencing technology has facilitated unprecedented advancements in microbiome research. This methodology allows for the rapid and efficient analysis of large-scale samples, thereby elucidating the diversity and composition of both gut and tumor microbiomes (75, 76). Through the application of whole genome sequencing, 16S rRNA gene amplification sequencing, and metagenomic studies, researchers have been able to investigate the associations between specific bacterial populations and tumor metastasis. By integrating these sophisticated sequencing technologies, a comprehensive understanding of the microbiome’s role can be achieved. For instance, research has demonstrated that gut microbiota can affect the effectiveness of cancer therapies and the broader tumor microenvironment, underscoring the significance of microbial diversity in cancer prognosis (77). Furthermore, the identification of specific bacterial taxa linked to various cancer types, including CRC and esophageal squamous cell carcinoma, underscores the potential of microbiome analysis as both a diagnostic tool and a therapeutic target (78, 79). Through the application of metagenomic methodologies, researchers are able to elucidate the functional capabilities of microbial communities, thereby offering insights into their roles in tumor biology and metastasis (80, 81).

This comprehensive approach not only enhances our understanding of cancer biology but also informs the development of microbiome-based therapeutic interventions for cancer. As the field of microbiome research progresses, the interactions between microbial communities and cancer may unveil novel therapeutic targets and strategies for managing tumor metastasis.

### 5.2 Exploration of innovative therapeutic strategies

In recent years, bacterial genetic engineering has emerged as a significant advancement in cancer therapy. Utilizing synthetic biology, researchers have developed methodologies to engineer bacteria capable of identifying and eradicating tumor cells. A frequently employed engineered bacterium is Escherichia coli Nissle 1917. Certain researchers have modified this bacterium to function as a temperature-sensitive entity, which can be activated through focused ultrasound to facilitate sustained tumor treatment (82). Moreover, recent studies have successfully engineered Escherichia coli to convert ammonia within tumors into L-arginine, thereby augmenting the anti-tumor efficacy of ICPI (83). Concurrently, other investigations are examining the potential of bacteria as drug delivery vectors, which can facilitate the targeted release of anti-cancer agents within the tumor microenvironment, thereby enhancing the specificity and efficacy of these therapeutic agents (84). This cutting-edge research holds promise for introducing novel perspectives and methodologies in cancer treatment.

The integration of bacterial agents with tumor immunotherapy represents a significant area of research interest. Certain bacterial species have the capacity to activate the host immune system, thereby augmenting the effectiveness of immunotherapeutic interventions. For instance, specific bacterial taxa, such as Bifidobacterium pseudolongum, Lactobacillus johnsonii, and Olsenella species, have been demonstrated to enhance the efficacy of ICPIs, facilitating improved recognition and destruction of cancer cells by the immune system (85). Research has demonstrated that specific gut bacteria, including Bifidobacterium and Ackermannia, have the capacity to stimulate the immune system by promoting T cell activation and augmenting cytokine production, which are essential for immune signal transduction (23, 86, 87).

In summary, elucidating the relationship between bacteria and the immune system presents novel opportunities for advancing cancer immunotherapy. This suggests that modulating the microbiome could be a promising strategy to enhance treatment outcomes for cancer patients.

### 5.3 Ethics and safety considerations

As bacteria-based therapeutic strategies advance towards clinical implementation, it is imperative to meticulously address ethical considerations and conduct comprehensive safety assessments. The utilization of genetically modified bacteria presents potential biosafety concerns, including possible effects on the environment and the human microbiome. Concurrently, ethical issues such as informed consent, privacy, and the equitable distribution of treatment warrant careful attention. In the development of novel therapeutic interventions, it is imperative to institute a robust ethical review framework to guarantee research transparency and safeguard patient interests. Furthermore, the implementation of thorough clinical trials and safety evaluations is essential to advance bacteria-based treatment strategies that minimize risk while ensuring the efficacy of new therapies and the safety of patients.

The intersection of microbiome research and tumor metastasis has emerged as a significant area of focus within cancer research. Empirical studies have demonstrated a close association between the bacterial composition of the tumor microenvironment and the risk of tumor metastasis. By analyzing the microbiome of patients, researchers aim to identify specific bacterial taxa as potential biomarkers, thereby facilitating early detection and personalized therapeutic strategies. Nonetheless, the practical application of these findings is hindered by challenges such as individual variability and the inherent complexity of the microbiome. These factors may impact the reproducibility and relevance of study outcomes, complicating their clinical translation. Furthermore, effectively integrating these research insights into clinical practice, alongside reconciling conventional treatment paradigms with emerging technologies, represents a critical challenge that must be addressed in future research endeavors.

Despite advancements in emerging technologies such as microbial engineering, which includes sequencing and bacterial innovations offering novel approaches to cancer treatment, their practical application continues to encounter numerous challenges. These challenges encompass the high costs associated with these technologies, the complexity of data processing, and the need for regulatory compliance. Moreover, ethical and safety evaluations are critical when implementing new therapies. For instance, while probiotic therapies have the potential to modulate the microbiome and enhance immune responses, ensuring their long-term safety for patients remains imperative. Consequently, the successful implementation of these technologies necessitates multidisciplinary collaboration, fostering comprehensive research in relevant fields, developing viable solutions, and undertaking rigorous clinical trials. This approach is essential to ensure that these innovative technologies can be effectively integrated into real-world settings, ultimately offering improved treatment options for cancer patients.

## 6 Discussion

Bacteria exhibit a multifaceted and significant influence on the process of tumor metastasis, demonstrating a clear dual role. On one hand, certain bacterial species can facilitate the metastasis and invasion of tumor cells by enhancing inflammatory responses, modifying the tumor microenvironment, and modulating the immune response. Conversely, specific bacteria and their metabolites have the potential to inhibit tumor growth, indicating that the role of bacteria in tumor development is not exclusively detrimental. This dual effect offers a novel perspective, implying that bacteria and their associated mechanisms could emerge as innovative targets for therapeutic intervention.

In the future, the industry ought to intensify the comprehensive investigation of the biological characteristics of bacteria, with particular emphasis on elucidating the mechanisms underlying their role in the regulation of tumor metastasis. The exploration of bacteria-tumor cell interactions should be further integrated with high-throughput techniques and bioinformatics to effectively address the complexity and uncertainty inherent in current research. Furthermore, multidisciplinary collaboration is anticipated to facilitate significant advancements in tumor treatment through the development of novel therapeutic strategies, such as bacterial combined immunotherapy and bacteria-based genetic engineering therapy. Overall, the investigation of bacteria as a regulatory factor in tumor metastasis is projected to offer innovative insights and directions for more precise and effective cancer therapies in the future.
